# Gestational Smoking and Hypertension as Predictors of Working Memory Functioning in Childhood Attention-Deficit/Hyperactivity Disorder

**DOI:** 10.3389/fpsyg.2020.01950

**Published:** 2020-09-11

**Authors:** Enitan T. Marcelle, Mercedes T. Oliva, Stephen P. Hinshaw

**Affiliations:** ^1^Department of Psychology, University of California, Berkeley, Berkeley, CA, United States; ^2^Department of Psychiatry, University of California, San Francisco, San Francisco, CA, United States

**Keywords:** attention-deficit/hyperactivity disorder, working memory, smoking, hypertension, prenatal, perinatal

## Abstract

Attention-deficit/hyperactivity disorder (ADHD) is a neurodevelopmental condition affecting between 5 and 8% of all children and adolescents, characterized by impairing levels of inattention, hyperactivity, and impulsivity. Existing cognitive models of ADHD have placed working memory (WM) deficits at the core of ADHD and suggest that primary WM deficits may also underlie the additional deficits associated with the condition. Although not all children with ADHD show WM deficits, those with such deficits have been found to have worse functional outcomes when compared to their diagnosed peers with typical WM functioning. Even so, contributors to the variability of impaired WM functioning seen within this population remain unknown. In the present study, we examined the association between two known prenatal and perinatal risk factors for impaired cognitive functioning – gestational smoking and hypertension – in three independent samples of children and adolescents with ADHD (samples varied with respect to sample size and WM measurement procedures). Contrary to hypotheses and existing literature, presence of high blood pressure during pregnancy was unexpectedly found to be a positive predictor of offspring WM capacity in one of three samples (a sample of all girls with ADHD). Implications and considerations for future research are discussed.

## Introduction

Attention-deficit/hyperactivity disorder (ADHD) is a commonly occurring behavioral condition characterized by developmentally extreme patterns of inattention, hyperactivity, and impulsivity (American Psychiatric Association, 2013; Polanczyk et al., 2014). Individuals diagnosed with ADHD demonstrate deficits across several cognitive (e.g., motivation, memory, processing speed) and functional (e.g., educational attainment and interpersonal relationships) domains. Furthermore, the condition is linked with increased rates of many psychiatric conditions (e.g., depression, anxiety, and conduct problems; Shaw-Zirt et al., 2005; Loe and Feldman, 2007; Brown, 2013; Antshel et al., 2014; Hinshaw, 2018). A clear pathogenesis is unknown, largely due to the cognitively and behaviorally heterogeneous presentation of this condition (Nigg et al., 2005; Hinshaw, 2018).

ADHD is a complicated constellation of commonly co-occurring and covarying symptoms, and efforts to optimize prevention and intervention in the absence of known biological mechanisms have focused on elucidating underlying cognitive mechanisms. This search has led to the development of several cognitive models of ADHD, including a functional working memory (WM) model. WM is the ability to store and manipulate mental information (Baddeley and Hitch, 1974; Baddeley, 1992). The WM model of ADHD places WM impairment at the core of observed deficits in ADHD (Kofler et al., 2008; Alderson et al., 2010). WM impairment is a common feature of ADHD (Martinussen et al., 2005; Huang-Pollock and Karalunas, 2010; Kasper et al., 2012); furthermore, WM deficits predict hyperactivity, impulsivity, social difficulties, and academic difficulties (Kofler et al., 2008; Alloway, 2009; Alderson et al., 2010; Kofler et al., 2010; Miller and Hinshaw, 2010; Kofler et al., 2014). Up to 62% of children with ADHD display WM deficits (Healey and Rucklidge, 2006; Mattfeld et al., 2016; Kofler et al., 2019); these children frequently show pronounced impairments across time and domains (Halperin et al., 2008). Importantly, a longitudinal study conducted by Karalunas et al. (2017) found that improvement in WM (but not in other related cognitive functions) predicted ADHD symptom remission across early and middle childhood in both parent and teacher reports.

Despite the known negative outcomes associated with impaired WM functioning in ADHD, much less is known regarding risk factors for WM impairment itself. Expanding the understanding of predictors of WM deficiency is an issue of important public health concern, given research demonstrating relations between WM deficits and negative social outcomes such as reduced school achievement (Gathercole and Pickering, 2000) and increased behavioral difficulties in childhood (Aronen et al., 2005). Furthermore, improvement in predictive ability should allow for the development of tailored and targeted intervention, potentially mitigating associated negative outcomes. Research points to the prenatal period as one of the most important and most vulnerable periods of brain development (Davison and Dobbing, 1966; Dobbing, 1990, 2008; Giussani, 2011). Identifying prenatal contributors to atypical cognitive functioning would enable early-life detection of at-risk populations. Leading candidate prenatal and perinatal predictors (of neurodevelopmental disorder broadly and of ADHD symptoms and diagnosis specifically) include prenatal tobacco exposure (PTE) as well as hypertensive disorders of pregnancy (Rodriguez and Bohlin, 2005; Silva et al., 2014; Huang et al., 2018; Maher et al., 2018). These risk factors are associated with cognitive impairment in offspring and also represent the most common substance used (tobacco) and disorder (hypertension), respectively, during pregnancy (Weitzman et al., 2002; Leeman and Fontaine, 2008).

Approximately 16% of women smoke while pregnant, making tobacco the most frequently used teratogen believed to impair fetal brain development (United States Department of Health and Human Services et al., 2012). Evidence of its effect on brain development is mixed, with some studies reporting nonsignificant relations between PTE and cognitive impairment after adjusting for variables such as socioeconomic status (SES), home environment, and mother’s intelligence or education (Baghurst et al., 1992; Kafouri et al., 2009). Yet, other works suggest a twofold to fourfold increase in ADHD diagnosis among offspring whose mothers smoked during pregnancy even after appropriate covariates are applied (Fried et al., 1998; Ernst et al., 2001; Cornelius and Day, 2009; Thapar et al., 2009; Ekblad et al., 2015). An inverse relation has also been shown between (a) PTE and (b) offspring WM and executive functioning impairment (Roy, 2002). Specific pathways underlying this association remain unclear (Thapar et al., 2009) but may include structural changes in the brain, compromised neuronal maturation (Roy, 2002), and alterations in dopaminergic and serotonergic systems (Ernst et al., 2001), all of which have been associated with ADHD and WM impairment (Volkow et al., 2005).

Up to 22% of women suffer from high blood pressure while pregnant, making hypertensive disorders of pregnancy one of the most common pregnancy complications (Yücesoy et al., 2005; Leeman and Fontaine, 2008). Despite mixed evidence, hypertensive disorders of pregnancy have also been associated with increased risk of offspring neuropsychological impairment, including ADHD diagnostic status and WM impairment (Pohlabeln et al., 2017; Maher et al., 2018). Two year olds born to mothers with preeclampsia (a pregnancy complication characterized by high blood pressure) demonstrated impaired attention when compared to their peers (Korkman et al., 1994). Much more recently, Pohlabeln et al. (2017) found that offspring of mothers who experienced pregnancy-induced hypertension were two times more likely to later be diagnosed with ADHD, whereas related common disorders of pregnancy such as proteinuria and gestational diabetes posed no increased risk. Ratsep et al. (2016) found that offspring of mothers with preeclampsia exhibited specific impairments in WM, but deficits in areas of cognitive functioning such as inhibition and executive functioning were not detected.

Mechanisms explaining the relation between hypertensive disorder of pregnancy and impaired fetal neurodevelopment are not yet well understood, but may include fetal malnutrition and intrauterine growth restriction, both of which can occur as a result of placental dysfunction associated with maternal hypertension. Fetal exposure to hypoxia occurring as a result of gestational hypertension has also been suggested to affect fetal brain development (Wade and Jenkins, 2016). Animal studies examining the effect of preeclamptic pregnancy on offspring neurodevelopment have shown disrupted neurogenesis (Liu et al., 2016). However, further work elucidating these mechanisms is needed.

We seek to expand understanding of the etiology of WM deficits within ADHD through different samples. Extant literature has shown the relation between maternal smoking and hypertension with ADHD diagnosis, specifically, and WM, broadly. However, we know of no work examining predictors of WM impairment within the diagnostic category. To this end, we examine the association between these candidate prenatal risk factors and the later WM functioning of ADHD-diagnosed offspring. We hypothesize that presence of these risk factors during the prenatal period will predict decreased offspring WM capacity.

## Materials and Methods

### Participants

Our sample consists of 140 girls with ADHD (combined and inattentive presentations) participating in the Berkeley Girls with ADHD Longitudinal Study (BGALS), a broader study (*N* = 228) investigating the longitudinal developmental trajectory of girls with and without ADHD. Given limited attention given in research to girls with ADHD (Quinn, 2005), we believe that investigating these processes in girls specifically is warranted. Because the aim of the present work is to investigate predictors of WM impairment specifically in ADHD, associations in the typically developing sample were not examined. The ADHD subsample (*N* = 140) ranged from 6 to 12 years old (mean = 9.6 years, SD = 1.7) at baseline (the sample was ascertained from 1997 to 1999). The sample is ethnically diverse, with 56% Caucasian, 28% African American, 11% Latina, 4% Asian American, and 1% Native American, with household incomes ranging from less than $10,000 to greater than $75,000, with an average of $50,000 to $60,000 (Table 1). Participants were recruited from the San Francisco area through mental health clinics, pediatricians, schools, and through advertisements to participate in research summer programs. The study was approved by the UC Berkeley Institutional Review Board, and informed consent/assent from parents and girls was obtained prior to participation. ADHD diagnostic status was determined using the Diagnostic Interview Schedule for Children (DISC) 4.0 (fourth edition; Shaffer et al., 2000) and the Swanson, Nolan, and Pelham Rating Scale (fourth edition; *SNAP-IV*; Swanson et al., 2001). At baseline, 93 girls met the criteria for ADHD combined type, and 47 for ADHD inattentive type. Seventy-one percent of girls with combined-type ADHD also met the criteria for oppositional defiant disorder (ODD), 26.9% also met the criteria for conduct disorder (CD), and 31.1% also met the criteria for an anxiety disorder, 46.8% of girls with inattentive-type ADHD also met the criteria for ODD, 10.6% also met the criteria for CD, and 19.1% also met the criteria for an anxiety disorder.

**Table 1:** Berkeley Girls with ADHD Longitudinal Study (BGALS) sample characteristics.

Characteristics	BGALS sample (*n* = 140)
Combined type	93 (66.4)
Inattentive type	47 (33.6)
Age at W1, years	9.6
Age at W3, years	19.7
Maternal age at child’s birth, years	27.93
SES	−0.07 ± 0.83
WM capacity	−0.52 ± 1.69
PIQ	99.43 ± 13.90
White	79 (56.4)
Nonwhite	60 (42.9)
High blood pressure	20 (14.3)
Tobacco use	27 (19.3)

Data are shown as mean ± SD or n (%).

Participants were subsequently invited to partake in longitudinal follow-up studies at 5 (wave 2: Hinshaw et al., 2006), 10 (wave 3: Hinshaw et al., 2012), and 16 (wave 4: Owens et al., 2017) years following baseline data collection. Retention was 92% at wave 2, 95% at wave 3, and 93% at wave 4. For additional information on this sample and its recruitment procedures, see Hinshaw (2002).

### Measures

#### Criterion Variable: Verbal Working Memory Functioning

Verbal WM capacity at wave 3 was used to determine WM functioning. WM capacity was determined by summing standardized Wechsler Abbreviated Scale of Intelligence (WASI) Letter Number Sequence (LNS) and Digit Span Backward (DSB) scores (standardized by a *z* score for each individual based on ADHD sample mean and standard deviation). This variable was calculated and used instead of the working memory index (WMI) provided by the WASI in order to avoid confounding WM with sustained attention. That is, the WMI includes a summation of performance on the Digit Span Forward (DSF) task plus performance on LNS and DSB. Given literature demonstrating that DSF is a measure of sustained attention, separable from WM (Gerton et al., 2004), we opted to calculate a new measure more accurately tapping relevant mental storage and manipulation abilities. Mean and standard deviation of this calculated variable can be found in Table 1. Data from wave 3 (17–23 years, mean age = 19.7 years) were chosen in order to assess WM functioning at the neuromaturational time point at which this capacity is likely to be at its maximum, as relevant literature suggests that WM capacity peaks by the early to mid-20s (Brockmole and Logie, 2013).

#### Predictor Variables/Risk Factors: Maternal Smoking and Maternal High Blood Pressure During Pregnancy

These predictor variables were assessed *via* retrospective maternal report data collected during baseline evaluation. Existing literature has demonstrated high reliability and validity of retrospective prenatal and perinatal event report (Owens and Hinshaw, 2013). In the present sample, mothers indicated whether they smoked cigarettes or had high blood pressure or toxemia while pregnant. Each variable was dichotomously scored.

#### Covariates

Selected covariates included SES, race, maternal age during pregnancy, and offspring IQ. SES, race/ethnicity of the child, and maternal age during pregnancy were selected as covariates based on research demonstrating association between these variables and the presence of smoking and high blood pressure during pregnancy (Kramer et al., 2000; Tanaka et al., 2007; Poon et al., 2010). SES was calculated using mother’s retrospective self-report of her own education (rated on a six-point scale) and pretax family income (rated on a nine-point scale). Scores were then standardized and averaged to create the SES variable. Offspring IQ was selected based on research demonstrating association between WM functioning and IQ (Conway et al., 2003). This covariate was measured using the Performance IQ (PIQ) provided by the WASI, which provides a measure of IQ based on perceptual organization and processing speed abilities and does not take WM functioning into account.

### Data Analytic Plan

All statistical analyses were performed with SPSS, version 25 (IBM Corp., 2017). First, an independent-samples *t*-test was conducted to determine the relation between presence or absence of the two risk variables and offspring WM. Effect size was calculated using Cohen *d*. When significant differences were found, we performed multiple regressions to address potential effects of the previously identified variables, including the covariates of interest, allowing us to specify prediction.

## Results

Table 1 displays characteristics of our sample. About 14.3% of mothers reported having high blood pressure or toxemia during pregnancy, and 19.3% reported smoking tobacco during pregnancy.

Children of hypertensive pregnancies had significantly increased WM capacity (mean = 0.20, SD = 1.72) compared to children of nonhypertensive pregnancies (mean = −0.72, SD = 1.68) [*t*(101) = −2.1, *p* = 0.038, *d* = 0.54] (Table 2). Given these results, we conducted regression analyses to assess the predictive contribution of high blood pressure during pregnancy to offspring’s WM, taking into account relevant covariates. Presence of high blood pressure during pregnancy contributed to the variance in offspring WM capacity (*β* = 1.19, *p* = 0.008), but SES, race/ethnicity, and maternal age during pregnancy did not significantly contribute. We, therefore, reconducted regression analysis using the most efficient model, testing the relation between high blood pressure during pregnancy and offspring WM capacity, adjusting for offspring PIQ (*β* = 1.18, *p* = 0.006, *R*^2^ = 0.161; Table 3). Specifically, both PIQ and maternal high blood pressure were significant predictors; hypertension continued to predict significant variance with PIQ included in the model. *Post hoc* analyses were conducted to examine relation between hypertensive pregnancy and offspring WM capacity separately in combined-type and inattentive-type samples. Sixteen percent (*n* = 15) of girls with combined-type ADHD and 8% of girls with inattentive-type ADHD (*n* = 5) had mothers who endorsed experiencing hypertension during pregnancy. Diagnostic status did not significantly contribute to variance in offspring WM capacity (*p* = 0.10), and *post hoc t*-tests did not reveal significant differences in WM capacity or presence of high blood pressure during pregnancy by group.

**Table 2:** Offspring working memory (WM) capacity by hypertensive pregnancy status (BGALS).

	Hypertensive pregnancy		Nonhypertensive pregnancy		*t*-test
	Mean	SD	Mean	SD	
WM capacity	0.20	1.72	−0.72	1.68	−2.1^*^

* *p* < 0.05.

**Table 3:** Summary of regression analysis for variables predicting offspring WM capacity (BGALS sample).

Variable	Model 1			Model 2		
	*B*	SE *B*	*p*	*B*	SE *B*	*p*
WISC PIQ	0.38	0.012	0.002	0.043	0.011	0.000
High blood pressure during pregnancy				1.18	0.417	0.006
Adjusted *R*^2^	0.085			0.144		
*F*	10.463			9.597		

Second, WM capacity of children whose mothers reported smoking tobacco during pregnancy (mean = −0.54, SD = 1.59) did not differ significantly from children whose mothers did not report smoking during pregnancy (mean = −0.53, SD = 1.73) [*t*(109) = 0.027, *p* = 0.979, *d* = 0.01] (Table 4). We did not follow up this nonsignificant test with regression analyses.

**Table 4:** Offspring WM capacity by prenatal tobacco exposure (PTE) status (BGALS).

	Prenatal tobacco exposure		No prenatal tobacco exposure		*t*-test
	Mean	SD	Mean	SD	
WM capacity	−0.54	1.59	−0.53	1.73	0.027

### *Post hoc* Analysis With Independent Samples

Findings from our first study failed to support either of our initial hypotheses. In fact, contrary to our hypothesis, results demonstrated a positive association between high blood pressure during pregnancy and WM capacity of the child. Results also demonstrated a nonsignificant relation between PTE and WM capacity. Given these unexpected results, we conducted *post hoc* analyses using two large and independent samples of children with ADHD in order to guard against reporting incidental, spurious, or sample-specific findings. Thus, we examined associations between previously identified prenatal risk factors (PTE and blood pressure status) and offspring WM capacity in two additional longitudinal samples. These two samples – Halperin Research Group sample (Rajendran et al., 2015) and Multimodal Treatment Study of ADHD (MTA) sample (MTA Cooperative Group, 1999) – along with subsequent analyses, are described below.

### Methods: Halperin Sample

#### Participants

The sample consists of a subsample of 98 individuals evaluated to have ADHD in preschool (aged 3–4 years, mean = 4.36, SD = 0.46). Diagnostic status was determined *via* the Kiddie Schedule for Affective Disorders and Schizophrenia: Present and Lifetime Version (Kaufman et al., 1997). All participants with ADHD met the criteria for combined type at baseline. Information on comorbid conditions was not reported in this sample. The sample consists of 76.1% male (23.9% female) individuals and is ethnically diverse, including 15.9% African American, 59.4% Caucasian, 35.5% Hispanic, and 18.8% mixed or other ancestry-identifying individuals (Table 5). Individuals were reassessed annually over 6 years. More information can be found in Rajendran et al. (2015).

**Table 5:** Multimodal treatment study of ADHD (MTA) and Halperin sample characteristics.

	MTA sample (*n* = 579)	Halperin sample (*n* = 98)
Age, years	8.5 ± 0.8	8.6 ± 0.32
Male	465 (80.3)	75 (76.1)
Female	114 (19.7)	23 (23.9)
White	353 (61)	58 (59.4)
Nonwhite	226 (39)	40 (40.6)
High blood pressure	67 (12.5)	4 (4.2)
No. tobacco	132 (23.9)	26 (27.4)

Data are shown as mean ± SD or n (%).

### Measures

#### Criterion Variable: Verbal Working Memory Functioning

Mirroring study 1 procedures, WM capacity was determined by summing the standardized LNS and DSB scores from the Wechsler Intelligence Scale for Children (WISC-IV). This information was collected when participants were aged 7–9 years (mean = 8.6, SD = 0.32). Mean WM capacity of offspring was −0.0026 (SD = 1.69).

#### Predictor Variables/Risk Factors: PTE and Maternal High Blood Pressure During Pregnancy

Predictor variables were assessed *via* retrospective maternal report data collected during baseline evaluation. Mothers were asked to indicate whether they smoked cigarettes or had preeclampsia while pregnant.

### Data Analytic Plan

All statistical analyses were performed with SPSS, version 25 (IBM Corp., 2017). Independent-samples *t*-tests were again utilized to determine group differences in WM capacity based on presence or absence of candidate prenatal factors. Effect size was calculated using Cohen *d*. When significant differences were found, regressions were performed to assess individual contribution of predictor variables to WM capacity.

### Results

Results did not show a significant difference in WM capacity between children of hypertensive pregnancies (4.2%) and children of nonhypertensive pregnancies: *t*(93) = 1.24, *p* = 0.219, *d* = 0.81. Results also did not show a significant difference in WM capacity between children whose mothers reported smoking tobacco (27.4%) and children whose mothers did not report smoking tobacco during pregnancy: *t*(92) = 0.81, *p* = 0.42, *d* = 0.19.

### Method: MTA Sample

#### Participants

This sample consists of 579 individuals with ADHD (all combined-type) participating in the MTA. Individuals participating in this study (aged 7–9 years, mean = 8.5 years, SD = 0.8) were recruited from areas around the United States and Canada for a randomized treatment study. ADHD diagnostic status was determined using the Diagnostic Interview Schedule for Children, Parent Report, version 3.0 (DISC-P; Shaffer et al., 1996). The sample consists of 80% male (20% female) and 61% white (39% nonwhite) individuals. Thirty-two percent of the overall sample met the criteria for ADHD and ODD/CD, 13% met the criteria for ADHD and an anxiety disorder, and 26% met the criteria for ADHD, ODD/CD, and an anxiety disorder. More information about subject characteristics can be found in MTA Cooperative Group (1999).

### Measures

#### Criterion Variable: Verbal Working Memory Functioning

WISC-III Digit Span scores collected during screening were used to measure WM capacity. In this sample, Digit Span data were entered as a cumulative score (i.e., forward, backward, and sequencing) and were not separated by subtest. As such, separating DSF (sustained attention) performance from backward and sequencing (WM) performance was not possible. The mean Digit Span scaled score was 8.81 (SD = 3.16).

#### Predictor Variables/Risk Factors: PTE and Maternal High Blood Pressure During Pregnancy

Predictor variables were assessed *via* retrospective maternal report data collected during initial/baseline evaluation. Mothers were asked to indicate whether they did or did not have high blood pressure during pregnancy and whether they did or did not smoke during pregnancy.

### Data Analytic Plan

All statistical analyses were performed with SPSS, version 25 (IBM Corp., 2017). Independent-samples *t*-tests were again utilized to determine group differences in WM capacity based on presence or absence of prenatal factors (high blood pressure or tobacco use). Effect size was calculated using Cohen *d*. When significant differences were found, regressions were performed to assess individual contribution of predictor variables to WM capacity.

### Results

Results did not show a significant difference in WM capacity between children of hypertensive pregnancies (12.5%) and children of nonhypertensive pregnancies: *t*(320) = 0.88, *p* = 0.38, *d* = 0.17. Results also did not show a significant difference in WM capacity between children whose mothers reported PTE (23.9%) and children whose mothers did not report PTE: *t*(330) = 1.32, *p* = 0.187, *d* = 0.18.

## Discussion

Our aim was to examine the relation between two core prenatal risk factors – maternal high blood pressure and smoking during pregnancy – and WM functioning in offspring diagnosed with ADHD. We predicted that the presence of these risk factors would be independently associated with reduced offspring WM capacity. Findings in an initial, all-female sample did not support either hypothesis. In fact, contrary to our hypothesis, presence of high blood pressure during pregnancy predicted better offspring WM capacity, an association that remained significant after adjusting for relevant covariates such as offspring IQ. ADHD subtype was not found to affect this finding. It is important to note, however, that frequency of high blood pressure in our inattentive sample was very low, possibly leading to inability to reliably detect group differences. PTE was not predictive of offspring WM capacity. Given these unexpected findings, we examined the relation between prenatal risk factors and offspring WM capacity in two additional and independent samples of children diagnosed with ADHD. Neither maternal high blood pressure during pregnancy nor PTE was predictive of offspring WM capacity in either additional sample. However, frequency of high blood pressure was extremely low in one sample (4.2%, *n* = 4), even though the effect size was large (*d* = 0.8). This combination of low frequency (with limited ability to detect significant effects) and high effect size suggests that a significant relation between gestational hypertension and offspring WM may be detected in an adequately powered sample.

Presence of high blood pressure during pregnancy was found to be a positive predictor of offspring WM capacity in one of our three samples (BGALS sample), explaining 16.1% of the overall variance in WM capacity. This high *R*^2^ value is comparable to findings investigating the effect of known teratogens (e.g., fetal alcohol exposure) on later cognitive function (Jacobson et al., 1994; Sood et al., 2001). High blood pressure was not a significant predictor of offspring WM capacity in either the BGALS or the two additional samples.

We hypothesize that variability in age across samples (mean age_BGALS_ = 19.7 years, mean age_Halperin_ = 8.6 years, mean age_MTA_ = 8.5 years) may partially contribute to observed differences in results. WM capacity is a frontal lobe function known to continue developing until approximately the age of 25 years (Brockmole and Logie, 2013). This trend has been replicated in the present BGALS sample as well. In fact, Gordon and Hinshaw (2019) showed that WM capacity in the BGALS sample continued to increase across development, plateauing at wave 3 (i.e., by an average age of 20 years). Literature examining the association between hypertensive disorders of pregnancy and offspring frontal functions such as behavioral inhibition has found gestational hypertension to be predictive of later impairment only after the age of 8 years, with the largest difference seen at age 14 years (Robinson et al., 2009).

Given variability in age across samples, we also conducted *post hoc* analyses examining relations between offspring WM and relevant prenatal conditions in wave 1 of BGALS data (mean = 9.6 years, SD = 1.7). Wave 1 results mirrored results found in wave 3 (i.e., presence of high blood pressure during pregnancy predicted better WM capacity, and PTE did not predict WM capacity). Although the mean age of participants in wave 1 is much closer to that of the Halperin and MTA samples, the difference in age still falls at a critical developmental point. Given literature suggesting differentiable WM performance between ages 6–8 and ages 9–10 years (Vuontela et al., 2003), it remains possible that the premature measurement of WM capacity (i.e., before the function had begun to mature) in the MTA and Halperin samples prevented us from detecting the influence of these prenatal risk factors on the development of this cognitive function.

Literature examining the effects of hypertension during pregnancy on fetal brain development remains inconclusive. Several theories may bear on mechanisms underlying an intuitively surprising positive association from the BGALS sample. Researchers operating from an evolutionary perspective propose that high blood pressure during pregnancy in humans may be adaptive, given the high frequency with which it occurs (Robillard et al., 2002). Thus, it is possible that, within limits, increases in blood pressure during pregnancy may increase blood flow and therefore nutrient transfer from mother to fetus (Gillon et al., 2014). However, in human studies, hypertensive medications may confound the effects of hypertension itself on fetal brain development. For example, Jong et al. (2010) revealed that offspring of mothers treated for gestational hypertension with labetalol were more likely to develop ADHD than either offspring of mothers treated with methyldopa or mothers without gestational hypertension. Additionally, different types of hypertensive disorder (e.g., chronic, gestational, preeclampsia, etc.) have been shown to differentially affect fetal brain development and associated neurodevelopmental outcomes (Maher et al., 2018). Several studies have shown hypertension to be a risk factor and preeclampsia a protective or nonsignificant factor with respect to fetal brain development (Robinson et al., 2009; Backes et al., 2011; Pohlabeln et al., 2017). Our binary variable construction did not allow for specification as to the type, timing, or treatment of the gestational high blood pressure; limiting broad implications are necessitating real caution in inferring any kind of protective link between maternal hypertension and enhanced offspring WM. In all, the relation between high blood pressure during pregnancy and children’s later WM capacity merits further consideration. Future research will aim to examine the differential effects of type and extent of hypertension on WM capacity of offspring. Additional questions include the mechanism by which these risk factors influence neurodevelopmental outcomes.

Null findings surrounding association between presence of fetal tobacco exposure and offspring WM capacity were replicated across three independent samples. It may well be that environmental factors such as length and timing of exposure, above and beyond mere presence, drive potential teratologic effects of the exposure (Fried et al., 1998; Ernst et al., 2001; Cornelius and Day, 2009; Thapar et al., 2009; Ekblad et al., 2015). For example Fried et al. (1998) found a dose-dependent negative association between cognitive functioning and PTE. Animal studies have suggested that nicotine exposure during late gestation only may increase the risk of ADHD-like symptoms in offspring (Thomas et al., 2000). Furthermore, *via* a design using genetically related and unrelated surrogate mothers who smoked during pregnancy, Thapar et al. (2009) proposed that inherited confounds (e.g., parental ADHD status) and not maternal smoking itself may drive associations between PTE and cognitive impairment (we were unable to covary maternal WM function). Importantly, these null findings may also be explained by variability in the causes of WM deficits themselves and/or by risk factors for WM deficits that share common pathways with gestational smoking or hypertension, such as premature birth. Future work may seek to utilize comprehensive medical record data in order to adequately covary/adjust for such risk factors. Taken as a whole, this literature highlights the many confounding variables outside of the scope of the present work. Future investigations should seek to investigate effect of tobacco exposure on WM capacity, taking into account relevant environmental factors like timing and frequency of use as well as maternal WM capacity.

The present study contains several important limitations. First, as noted above, the use of binary retrospective response data in all samples prevented investigation of the more nuanced effects of our risk factors of interest, including the effect of PTE on gestational blood pressure (Conde-Agudelo et al., 1999). Future investigation will benefit from the careful consideration of variable construction. Secondly, we had measures of verbal WM only. Given research demonstrating the separability of spatial and verbal WM (Shah and Miyake, 1996), future work would benefit from investigating differential effects of relevant risk factors on different areas of WM.

Despite limitations, this work presents an incremental step in elucidating the effects of prenatal conditions on the WM development of offspring. To our knowledge, this is the first study to examine the relation between prenatal risk factors and later WM functioning in childhood ADHD, and findings contribute to a growing body of work examining impact of prenatal risk factors on cognitive capacity. Future research will aim to investigate this complex relation utilizing more continuous measures of risk factor influence and more comprehensive (e.g., visual in addition to auditory) measures of WM functioning. Additional future directions include investigating the mechanisms by which these prenatal conditions may affect cognitive outcomes.

## Data Availability Statement

The raw data supporting the conclusions of this article will be made available by the authors, without undue reservation.

## Ethics Statement

The studies involving human participants were reviewed and approved by Institutional Review Board at the University of California, Berkeley. Written informed consent to participate in this study was provided by the participants’ legal guardian/next of kin.

## Author Contributions

EM: study design, data organization, data analysis, and drafting of manuscript. MO: data organization, data analysis, and drafting of manuscript. SH: data acquisition, data analysis tools, and drafting of manuscript. All authors contributed to the article and approved the submitted version.

### Conflict of Interest

The authors declare that the research was conducted in the absence of any commercial or financial relationships that could be construed as a potential conflict of interest.
